# Highly sensitive persons feel more emotionally lonely than the general population

**DOI:** 10.1038/s41598-025-87138-w

**Published:** 2025-01-21

**Authors:** Filip Meckovsky, Lukas Novak, Zdenek Meier, Peter Tavel, Klara Malinakova

**Affiliations:** OUSHI – Olomouc University Social Health Institute, Palacký University Olomouc, Univerzitni 244/22, 771 11 Olomouc, Czech Republic

**Keywords:** High sensitivity, Sensory processing sensitivity, Emotional loneliness, Social loneliness, Social isolation, Psychology, Risk factors

## Abstract

This study aimed to investigate the relationship between sensory processing sensitivity (SPS), emotional and social loneliness and social isolation. Data were collected from September 2022 to May 2023 on a sample of 3247 participants aged 18 to 80 (mean age = 31.9 years ± 13.2; 66.2% female). We measured SPS using the Sensory Processing Sensitivity Questionnaire (SPSQ), loneliness using the De Jong Gierveld Loneliness Scale (DJGLS), and social isolation, Neuroticism and Extraversion with the Big Five Inventory. Data analysis was performed using linear regression, binary logistic regression, the t-test, the Chi-square test and ANOVA. In our study, lower SPS was observed especially among men, pensioners and graduates of secondary vocational schools. SPS was associated with emotional loneliness (t = 4.276; b = 0.074; adjusted R2 = 0.181; *p* < 0.001), but no significant relationship was found between SPS and social loneliness. SPS is associated with higher emotional but not social loneliness or social isolation. Highly Sensitive Persons (HSPs) appear to have a higher need for intimacy and understanding in close relationships, which is essential to know for them, their friends, families and therapists.

**Supplementary Information:**

The online version contains supplementary material available at 10.1038/s41598-025-87138-w.

## Introduction

Over the past two decades, there has been a significant increase in scientific interest in sensory processing sensitivity (SPS)^1^. SPS has also gained considerable popularity among the general public; however, it is often misinterpreted due to a lack of relevant information^2^. SPS is described as a personality trait that has a neurobiological basis and is related to a higher reactivity of the nervous system^3,4^ and is moderately heritable^5^. SPS refers to sensory stimuli, such as smells, odours or light, as well as emotional and social stimuli^6^. It is defined by greater sensitivity to subtle stimuli and deeper information processing^7^. However, this sensitivity can lead to more frequent stimulation overwhelm^8^. According to recent findings, the authors lean towards a continuous distribution of the trait in the population, with about 31% being highly sensitive, 40% having medium sensitivity and 29% having low sensitivity^9,10^.

SPS differentially engages brain regions and processes information in a distinct way, which in turn is reflected in personality formation^3^. Given that SPS has been associated with neuroticism, openness to experience and introversion^2^, there is an ongoing debate regarding the extent to which it overlaps with existing theories of personality^11^, particularly Big Five model or the Regulative Theory of Temperament^12^. In particular, SPS overlaps with neuroticism, due to a shared tendency toward internalization and emotional instability, with introversion, due to increased shyness, and with openness to experience, due to an enhanced aesthetic sensitivity that is associated with a more open approach to new experiences^11^. Therefore, some researchers argue that SPS can be fully explained on the basis of Neuroticism and Openness to Experience^13^. However, follow-up research with larger samples agree that SPS represents a distinct personality trait unique of psychological adjustment and interpersonal sensitivities over and above personality traits^14,15^ .

SPS is also associated with increased emotional reactivity, which involves positive and negative emotions^16^. Highly Sensitive Persons (HSPs) are more responsive to experiencing positive emotions^17^ and are more easily induced into a cheerful mood^9^. They can benefit more from stimulating environments and positive events^16^, intervention programmes^18^ and psychotherapy^19^. Their greater responsiveness to external stimuli makes learning from situations easier, leading to their better handling in the future^6^. In addition, HSPs appear to be more empathetic and nurturing and can respond better to the needs of others^1^; they are also more creative^20^.

However, SPS has its disadvantages. HPSs are more susceptible to adverse childhood experiences and uncaring parenting^21^, which can have negative impacts on their well-being. Research has linked SPS to low life satisfaction^22^, negative emotions, including depression and anxiety^21,23,24^, difficulty regulating one’s emotions^25^, and higher levels of stress and symptoms of physical illness^26^. SPS, in the context of mental health, is associated with alexithymia, autism spectrum disorder symptoms^27^, mild cognitive impairment, dementia^28^ and attention deficit disorder^29^. Thus, HSPs account for up to half of patients receiving psychotherapy despite being a minority in the population^30^.

SPS has also been found to have a negative social impact, with links to shyness^23^, low self-esteem^31^ and social phobia^32^. HPSs may face stigma associated with being different from others and not being understood by society^1^, which can lead to feelings of loneliness and alienation from others. Research has not, as far as we know, addressed the area of social isolation and loneliness in HSPs^2,33^, despite the fact that loneliness is currently a worldwide problem^34^ with serious health consequences^35^ which affects more vulnerable groups^36^, including HSPs. Loneliness is defined as a negative subjective feeling of missing relationships, distinct from social isolation, representing an objective lack of relationships with others^37^. The only research carried out on the association of SPS and loneliness in the general population suggests that during the COVID-19 pandemic, HSPs were at greater risk of feeling loneliness^38^.

The above studies suggest a positive association between SPS and feelings of loneliness, but this association has not yet been adequately explored. Given greater emotional and social sensitivity and feelings of alienation from others, we expect HSPs to feel higher levels of loneliness compared to the general population. Therefore, this study aims to investigate the relationship between SPS and loneliness and social isolation.

## Methods

### Participants and procedure

The data was collected using an online questionnaire developed at our institution – the Social Health Institute, Palacký University Olomouc. The survey was distributed using a snowball technique from September 2022 to May 2023. Respondents were recruited through contacts of university students as an educational component of their coursework, rewarding them with academic credits.

Due to the online nature of the questionnaire, the following exclusion criteria were introduced to ensure high data quality: (1) missing responses to variables of interest; (2) filling in the questionnaire too quickly (under 26 min for a survey which generally lasted about 90 min); (3) providing identical answers to at least three questionnaires used in this study; (4) repeated completion of the questionnaire by one person. Altogether, 11,102 participants participated in the data collection, and 7855 were excluded due to the above-mentioned criteria. Therefore, the final sample comprised 3247 participants aged 18 to 80 (mean age = 31.9 years, SD = 13.2; 66.2% female).

Before the survey, participants received written information on the study’s goal, data confidentiality and anonymisation. Participation in the research was voluntary. Informed consent was obtained from all subjects and/or their legal guardian(s). They may, however, leave at any moment and without explanation. A pilot study was carried out among volunteers at Palacký University Olomouc before the main study. The study design was approved by the Ethics Committee of the Olomouc University Social Health Institute, Palacký University Olomouc (No. 2021/3). All experiments were performed in accordance with relevant guidelines and regulations.

### Measures

#### Sensory processing sensitivity

The Sensory Processing Sensitivity Questionnaire – SPSQ^38^ was used to measure SPS. The questionnaire comprises 16 items divided into two scales – a Sensory Sensitivity subscale (such as lights, sounds, smells, etc.) and Other Sensitivity subscale, which focuses on emotions and other life experiences (such as sudden changes, harmony in life, criticism, etc.). The respondents are instructed as follows: *Please indicate to what extent you think that compared to other people*,* you are sensitive to the following stimuli*. Possible answers range from 1 to 10 for each area, where 1 = *compared to others*, *I am not sensitive to them at all*; 5 = *about the same as the people around me*; and 10 = *much more sensitive than the people around me*. Final scores can range from 16 to 160, with higher values indicating greater sensitivity to stimuli. The total scale showed high internal consistency in our sample, with Cronbach’s alpha = 0.86, and the sensory sensitivity subscale showed Cronbach’s alpha = 0.75. For further statistical analysis, we adopted the approach of categorising the total SPS score^9^. We distinguished the scores obtained from the SPSQ into three categories: 16–64 = *low*, 65–112 = *medium* and 113–160 = *high* sensory sensitivity, and for the SPSQ sensory subscale, 8–32 = *low*, 33–56 = *medium* and 57–80 = *high* sensory sensitivity.

#### Loneliness

The De Jong Gierveld Loneliness Scale^39^ was used to measure perceived loneliness. The scale contains a total of 11 items which are divided into subscales of emotional (6 negatively formulated items) and social (5 positively formulated items) loneliness. Social loneliness refers to missing social networks, whereas emotional loneliness refers to a lack of intimate relationships^40^. For each item, an individual can answer 1 = *yes*, 2 = *more or less*, and 3 = *no*, which is recommended for online collections^37^. A point is then awarded for responses from these three categories if they indicate loneliness, including responses falling into the middle category of *more or less*. The total score ranges from 0 to 11, with higher scores corresponding to higher levels of loneliness. In our research, we used a version that was adapted for the Czech environment^41^. The scale showed high internal consistency in our sample, with Cronbach’s alpha = 0.83. For further statistical analysis, we dichotomised the scale scores, with scores of 0–2 = *not lonely* and scores of 3 or more = *lonely* for the overall, emotional and social scales^42^.

#### Social isolation

We used the following five questions (see Supplementary Materials S1) to measure social isolation, which is typically measured by social network breadth and interaction with close others^43^. The first two questions focused on the frequency of social interactions: *How often do you meet with* (a) *Close family members who do not live with you?* (b) *Friends?* With response options from 1 = *less than once a year or never* to 6 = *three or more times a week*. The following three questions focused on social network breadth: (c) *Please indicate the number of people you can contact for practical help*. (d) *How many really close friends do you have?* With response options ranging from 1 = *none* to 5 = *ten and more.* Moreover, last (e) *How many other friends do you have*? With response options ranging from 1 = *none* to 5 = *twenty and more*. Higher scores on each item correspond to lower social isolation. Since social isolation is not a homogeneous scale, we approached each item separately in the analyses and, therefore, there was no need to calculate Cronbach’s alpha. For further statistical analysis, we dichotomised the response items: meeting with family/friends: 1–3 = *infrequent* meetings; 4–6 = *frequent* meetings. People willing to help/close friends/friends: 1–3 = *small* social network; 4–5 = *large* social network.

#### Neuroticism, extraversion

To measure Neuroticism and Extraversion, we used the Neuroticism and Extraversion Subscale of the Big Five Inventory (John & Srivastava, 1999). Both subscales consist of 8 items, where the participant expresses a level of agreement with each statement on a 5-point Likert scale ranging from 1 = *strongly disagree* to 5 = *strongly agree*. The final scores range from 8 to 40, with higher scores corresponding to higher levels of Neuroticism and Extraversion. Both subscales showed high internal consistency in our sample, with Cronbach’s alpha (Neuroticism) = 0.87 and Cronbach’s alpha (Extraversion) = 0.86.

Sociodemographic characteristics, such as gender (male-female), age, family status, employment status and education level, were obtained by the questionnaire.

### Statistical analysis

In the first step, we described the sociodemographic characteristics of the study sample and the variables studied in each group. To compare the differences between the sociodemographic groups, we used a t-test for gender and family status and one-way ANOVA with post-hoc tests for the rest of the variables that included more than two categories. For the post-hoc analysis, we used Bonferroni correction. We used the chi-square test to compare frequencies for social isolation. To examine the effect of SPS (predictor) on loneliness (outcome), we used multivariate linear regression adjusted for age, gender, education, neuroticism and extraversion. We tested linear regression assumptions using the Durbin-Watson test for autocorrelation, multicollinearity (variance inflation factor) and normal distribution of residuals using a Q-Q plot. In the last step, we calculated the effect of SPS (predictor) on loneliness using binary logistic regression with categorical variables. For the logistic regression, we tested the multicollinearity criterion and outliers in the data.

All analyses were performed using the statistical software IBM SPSS version 21 (IBM Corp., Armonk, NY, United States).

## Results

Table 1 describes the sociodemographic characteristics of the study sample. A comparison of the sociodemographic groups in SPS showed significant differences (*p* < 0.05) in gender, employment status and education level, with the highest scores for women, those without employment and those with primary school and higher vocational or university a bachelor’s degree. Regarding the SPSQ total in employment status, people without jobs showed the highest scores, followed by people with jobs and the pensioners with the lowest scores. The same was true for the SPSQ sensory subscale, except when comparing people with and without jobs, where no significant difference was found. Compared to all other groups, people with secondary vocational schools showed the lowest SPS in the SPSQ total. In the SPSQ sensory subscale, there was a significant difference between secondary vocational and higher vocational or a university bachelor’s degree. Of the total sample, 617 (19%) were classified as high SPS, 2561 (78.9%) as medium and the remaining 69 (2.1%) as low sensitive. Similarly, according to the SPS sensory subscale, 434 (13.4%) were classified as high SPS, 2724 (83.9%) as medium and 89 (2.7%) as low sensitive.

**Table 1:** Descriptive statistics of the study sample and a sociodemographic comparison.

Sociodemographic group		SPSQ total	SPSQ sensory subscale
	N (%)	M (SD) p-value	M (SD) p-value
Total	3247 (100%)	97.55 (17.62)	46.97 (8.92)
Gender		*p* < 0.001	*p* < 0.001
1. Male	1099 (33.85%)	91.66 (17.22)	45.23 (8.99)
2. Female	2148 (66.15%)	100.57 (17.06)	47.86 (8.76)
Employment status		*p* < 0.001 (1–2**, 1–3**, 2–3***)	*p* < 0.001 (1–3***,2–3***)
1. With a paid job	1857 (57.19%)	96.86 (18.29)	46.87 (9.23)
2. Without a paid job	1283 (39.51%)	99.06 (16.45)	47.38 (8.41)
3. Disabled/old-age pensioner	107 (3.3%)	91.4 (17.48)	43.82 (8.8)
Education level		*p* < 0.001 (1–2***,2–3***,2–4***,2–5*)	*p* = 0.038 (2–4*)
1. Elementary school	170 (5.24%)	99.47 (19)	47.32 (9.9)
2. Secondary vocational	313 (9.64%)	93.45 (19.31)	45.64 (9.83)
3. Secondary graduation	1660 (51.12%)	97.9 (16.97)	47.11 (8.52)
4. Higher vocational or University bachelor	573 (17.65%)	98.53 (17.32)	47.39 (8.87)
5. College	531 (16.35%)	97.21 (18.16)	46.75 (9.25)
Family status		*p* = 0.600	*p* = 0.366
1. Married/partnership	1997 (61.5%)	97.68 (17.67)	47.08 (8.9)
2. Single/divorced/widow(er)	1250 (38.5%)	97.34 (17.56)	46.79 (8.95)

Notes: * *p* < 0.05; ** *p* < 0.01; *** *p* < 0.001.

A comparison of the low, medium and high sensitivity groups on loneliness and social isolation is shown in Table 2. The results reveal a significant difference in overall and emotional loneliness, where the high sensitivity group scored higher than the low and medium sensitivity groups. No significant difference was found in social loneliness. When comparing social isolation, there was a significant difference in the social network regarding people willing to help and friends, with the medium sensitivity group coming out with the highest representation and the low sensitivity group with the lowest representation. No significant difference was found between the sensitivity groups regarding interactions with family and friends or in the number of really close friends.

**Table 2:** Comparison of sensitivity groups in loneliness and social isolation.

Variable	Low sensitivity	Medium sensitivity	High sensitivity	Group differences
N (%)	69 (2.1%)	2561 (78.9%)	617 (19%)	
	M (SD)	M (SD)	M (SD)	
**Loneliness**				
1. Overall	4.28 (2.75)	4.62 (3.11)	5.15 (3.27)	*p* < 0.001 (1–2*, 1–3***)
2. Emotional	1.35 (1.77)	1.86 (1.96)	2.41 (2.08)	*p* < 0.001 (1–2***, 1–3***)
3. Social	2.93 (1.69)	2.76 (1.71)	2.74 (1.73)	n.s.

	Frequency N (%)	Frequency N (%)	Frequency N (%)	Group differences
**Social isolation**				
1. Meeting with family				*p* = 0.382
Infrequent interaction	11 (15.9%)	476 (18.6%)	128 (20.7%)	
Frequent interaction	58 (84.1%)	2085 (81.4%)	489 (79.3%)	
2. Meeting with friends				*p* = 0.323
Infrequent interaction	12 (17.4%)	374 (14.6%)	104 (16.9%)	
Frequent interaction	57 (82.6%)	2187 (85.4%)	513 (83.1%)	
3. People willing to help				*p* = 0.030
Small social network	52 (75.4%)	1654 (64.6%)	425 (68.9%)	
Large social network	17 (24.6%)	907 (35.4%)	192 (31.1%)	
4. Really close friends				*p* = 0.510
Small social network	60 (87.0%)	2101 (82.0%)	512 (83.0%)	
Large social network	9 (13.0%)	460 (18.0%)	105 (17.0%)	
5. Other friends				*p* = 0.020
Small social network	44 (63.8%)	1259 (49.2%)	341 (55.3%)	
Large social network	25 (36.2%)	1302 (50.8%)	276 (44.7%)	

Notes: * *p* < 0.05;  *** *p* < 0.001.

To determine the effect of SPS on overall, emotional and social loneliness, we used multivariate linear regression, presented in Table 3. The results showed that after controlling for age, gender, education, neuroticism and extraversion, there was no significant relationship between the SPSQ total and overall loneliness. However, there was a significant association between the SPSQ sensory subscale and overall loneliness, with beta = 0.042, adjusted R^2^ = 0.160. A higher SPSQ total score was associated with higher emotional loneliness, with beta = 0.074, adjusted R^2^ = 0.181. Regarding the SPSQ sensory subscale, there was a similar but weaker relationship with emotional loneliness. After adjusting for the variables, no significant relationship was found between SPS and social loneliness. In the crude effect, both scales (the SPSQ total and the sensory subscale) predicted higher overall and emotional loneliness but not social loneliness.

**Table 3:** Results of multivariate linear regression models assessing the effect of sensory processing sensitivity (predictors) on overall, emotional and social loneliness (outcomes), adjusted for age, gender, education, neuroticism and extraversion.

		Overall loneliness				Emotional loneliness				Social loneliness			
		Beta coefficient	Adjusted R^2^	Standard Error	t	Beta coefficient	Adjusted R^2^	Standard Error	t	Beta coefficient	Adjusted R^2^	Standard Error	t
SPSQ total	Crude	0.087***	0.007	0.003	4.997	0.149***	0.022	0.002	8.595	-0.013	0.000	0.002	-0.759
	Adjusted	0.032	0.160	0.003	1.833	0.074***	0.181	0.002	4.276	-0.027	0.086	0.002	-1.484
SPSQ sensory subscale	Crude	0.055**	0.003	0.006	3.135	0.090***	0.008	0.004	5.162	-0.004	0.000	0.003	-0.240
	Adjusted	0.042*	0.160	0.006	2.534	0.059***	0.179	0.004	3.618	0.008	0.086	0.003	0.464

Notes: * *p* < 0.05; ** *p* < 0.01; *** *p* < 0.001.

Significance value bold.

Table 4 shows the associations of the SPS groups with each social isolation area, adjusted for age, gender, education, neuroticism and extraversion. Compared to individuals with low sensitivity, individuals with medium and high sensitivity showed a 49% and 44% decrease in the odds of having a small group of friends, with similar results found for the SPSQ subscale. In addition, compared to the low sensitivity group, medium sensitive respondents showed a 47% decrease in the odds of having only a small group of people willing to help.

**Table 4:** Results of binary logistic regressions assessing the effect of sensory processing sensitivity (predictor) on social isolation (outcome), with not socially isolated being the reference category, adjusted for age, gender, education, neuroticism and extraversion.

		SPS group	Infrequent meet. family	Infrequent meet. friends	Small network help	Small network close friends	Small network friends
			Odds ratio (95% CI)	Odds ratio (95% CI)	Odds ratio (95% CI)	Odds ratio (95% CI)	Odds ratio (95% CI)
SPSQ total	Crude	Low	1	1	1	1	1
		Medium	1.20 (0.63–2.31)	0.81 (0.43–1.53)	0.60 (0.34–1.04)	0.69 (0.34–1.39)	0.59 (0.36–0.97)*
		High	1.38 (0.70–2.71)	0.96 (0.50–1.86)	0.72 (0.41–1.28)	0.73 (0.35–1.52)	0.70 (0.42–1.18)
	Adjusted	Low	1	1	1	1	1
		Medium	1.28 (0.66–2.47)	0.73 (0.41–1.52)	0.53 (0.30–0.95)*	0.58 (0.28–1.19)	0.51 (0.30–0.86)*
		High	1.54 (0.77–3.07)	0.93 (0.47–1.87)	0.58 (0.32–1.06)	0.57 (0.27–1.21)	0.56 (0.32–0.96)*
SPSQ sensory subscale	Crude	Low	1	1	1	1	1
		Medium	0.88 (0.52–1.49)	0.84 (0.48–1.47)	0.70 (0.43–1.12)	0.95 (0.54–1.67)	0.58 (0.37–0.90)*
		High	1.18 (0.67–2.07)	1.11 (0.61–2.04)	0.72 (0.43–1.20)	0.90 (0.49–1.64)	0.51 (0.31–0.81)**
	Adjusted	Low	1	1	1	1	1
		Medium	0.90 (0.53–1.53)	0.85 (0.47–1.54)	0.65 (0.40–1.07)	0.85 (0.48–1.52)	0.53 (0.33–0.83)**
		High	1.26 (0.71–2.24)	1.28 (0.68–2.41)	0.67 (0.40–1.14)	0.83 (0.45–1.55)	0.46 (0.28–0.76)**

Notes: *p < 0.05; ** p < 0.01. CI = confidence interval. Significance value bold.

## Discussion

This study examined the relationship between SPS, loneliness and social isolation in adults. When comparing sociodemographic characteristics, lower SPS values were found for men, pensioners and people in secondary vocational schools. Results showed that when controlling for age, gender, education, neuroticism and extraversion, SPS had a significant effect on emotional loneliness, with higher SPS corresponding to higher emotional loneliness. SPS had no significant effect on overall and social loneliness, except for the SPSQ sensory subscale, which showed an effect on overall loneliness. When assessing social isolation, we found that compared to medium and highly sensitive people, those with low sensitivity have fewer friends and people willing to help them. SPS had no significant effect on family meetings, meetings with friends or the number of really close friends.

Our results, consistent with previous studies^7,38,44,45^, showed that women score higher on the SPS scale than men. Moreover, the measurement invariance testing results showed a difference between men and women that cannot be explained by the measurement ability of the instrument^38^. One explanation for the gender difference tends to be attributed to Western culture, where the display of sensitivity in men may be perceived as weakness; therefore, men tend not to acknowledge it or mask this trait^7,44^. Trå et al.^45^ offered a second explanation. They found a difference in SPS between men and women even after controlling for the Big Five personality traits, which they explain from an evolutionary perspective as an adaptive advantage for women. This implies that the higher sensitivity of women cannot be fully explained based on a cultural hypothesis and that further investigation of other factors is needed.

Regarding education, the lowest SPS values were found for secondary vocational schools, which is in line with^38^, and they were significantly lower than every other level of education. One possible explanation is the higher representation of males than females in secondary vocational schools in Czechia^46^, but this did not prove to be the case in our sample. Another explanation is that secondary vocational education focuses mainly on manual work areas, whereas HSPs are described as empathetic and nurturing^1^ and would be more likely to be seen in the helping professions that typically require a high school education or higher.

Our results showed that even after controlling for age, gender, education, neuroticism and extraversion, HPSs feel more emotionally but not socially lonely. Despite the high correlation between emotional and social loneliness on the DJGLS scale, it seems that it is possible to distinguish between the emotional and social aspects of loneliness^47^, which appears to be a more accurate use of the loneliness scale in Czech society^41^. Therefore, we focus on explaining emotional and social loneliness separately rather than overall loneliness. One explanation may be that HSPs feel different from others and feel that they cannot be fully understood^1^. They tend to have a higher need for meaningful and deep relationships that they may not be getting. Therefore, they may have difficulty establishing or maintaining intimate relationships that require sharing deep emotions and thus feel more emotionally lonely. Although HSPs have a higher need for depth and quality of relationships, which results in emotional loneliness, they do not have a greater need for regular relationships in a broader social network, reflected in social loneliness. Furthermore our finding that SPS is associated with emotional loneliness despite controlling for neuroticism and extraversion, which appear to be the strongest predictors of loneliness from the Big Five Model^48^ contributes to the discussion that SPS represents a distinct personality trait.

Despite SPS being associated with shyness^23^, low self-esteem^31^ and social phobia^32^, it is not associated with higher social loneliness or social isolation. Conversely, it appears that people with low sensitivity have fewer friends and people willing to help them, which may be explained by the fact that high sensitivity is associated with a better ability to empathise with others and respond better to their needs^1^.

When analysing the relationship between SPS and loneliness, one must consider the attachment that may interfere with the relationship. High rates of SPS in adults are associated with insecure attachment^49^, which is characterised by uncertainty about one’s worth and problems with trusting others^50^. In the case of insecure attachment, a positive connection with loneliness was found; in contrast, a negative connection was found with secure attachment^51^. Given the positive relationship between SPS and insecure attachment and insecure attachment and loneliness, it is necessary to investigate further whether high SPS is related to greater emotional loneliness with attachment control, where attachment may be a potential moderating variable.

Our results show that people with higher SPS are more likely to experience a lack of intimacy and understanding in close relationships than the general population. This finding may be of primary importance for all HSPs, their families and loved ones to better understand their functioning in relationships. Further, since HSPs are estimated to make up about half of all clients in therapy^30^, it may be essential for all professional therapists to focus on improving the quality of functioning in close relationships. The findings may be valuable for parents and group leaders working with highly sensitive children, helping them choose appropriate activities and facilitating interactions with peers who share similar traits, creating a more supportive and understanding environment. Future research is recommended to explore the potential influence of attachment and childhood trauma on the relationship between SPS and loneliness and explore the relationship between SPS and loneliness with control of the remaining Big Five personality traits. Furthermore, future research could confirm the causality of our findings.

Our study is the first to focus on social and emotional loneliness in HSPs and controls for extraneous variables to eliminate confounding by other factors. Another strength is the large sample size, which helps minimise the influence of random errors and increases the precision of the results. In our research sample there were more women and a higher representation of younger and middle-aged people, compared to the national representative sample, which should be taken into account when generalising the results. Additionally, we utilised self-rating scales, which could be influenced by social desirability and the tendency for impression management. Another limitation is the significant p-value for the Durbin-Watson test in the case of emotional loneliness. However, the d-value was 1.91, autocorrelation = 0.04, which should not bias standard errors and thus p-values in model parameters. A final limitation is the use of a cross-sectional study from which causal inferences cannot be drawn.

In conclusion our results show that SPS is associated with higher emotional but not social loneliness or social isolation. Thus, HSPs appear to have a higher need for intimacy in close relationships, which they lack. In terms of ordinary social relationships, they interact like the general population. This finding is essential in light of increasing loneliness, which has negative health consequences, especially for at-risk groups, which HSPs represent. Finally, the present study adds relevant information about the SPS area, which is often misinterpreted by the general public.

## Electronic supplementary material

Below is the link to the electronic supplementary material.

Supplementary Material 1.

## Data Availability

The datasets generated during and/or analysed during the current study are available in the Open Science Frame work repository website: https://osf.io/6jxyu/?view_only=46ded50e388545e68404aa67f9de6fc3.
