# Supplementary material for: Highly sensitive persons feel more emotionally lonely than the general population
Source: Sci Rep. 2025 Jan 21;15:2707. doi: 10.1038/s41598-025-87138-w (PMC11751071; doi:10.1038/s41598-025-87138-w)
Supplement: Supplementary file 1 — Supplementary Material 1. [file 41598_2025_87138_MOESM1_ESM.pdf]

Supplement: Highly Sensitive Persons feel more emotionally lonely than the general population

Filip Meckovsky, Lukas Novak, Zdenek Meier, Peter Tavel, Klara Malinakova

Social isolation

A) *How often do you meet with close family members who do not live with you?*

Infrequent meetings:

1) less than once a year or never
2) once or twice a year
3) once every few months

Frequent meetings:

4) once or twice a month
5) once or twice a week
6) three or more times a week

B) *How often do you meet with friends?*

Infrequent meetings:

1) less than once a year or never
2) once or twice a year
3) once every few months

Frequent meetings:

4) once or twice a month
5) once or twice a week
6) three or more times a week

C) *Please indicate the number of people you can contact for practical help*

Small social network:

1) none
2) one to two
3) three to four

Large social network:

4) five to nine
5) ten and more

D) *How many really close friends do you have?*

Small social network:

1) none
2) one to two
3) three to four

Large social network:

4) five to nine
5) ten and more

E) *How many other friends you have?*

Small social network:

1) none
2) one to five
3) six to nine

Large social network:

4) ten to nineteen
5) twenty and more
